# Supplementary material for: Graphene Oxide Nanoparticles Having Long Wavelength Absorbing Chlorins for Highly-Enhanced Photodynamic Therapy with Reduced Dark Toxicity
Source: Int J Mol Sci. 2019 Sep 5;20(18):4344. doi: 10.3390/ijms20184344 (PMC6769858; doi:10.3390/ijms20184344)
Supplement: Supplementary file 1 [file ijms-20-04344-s001.pdf]

Supporting Information

Graphene Oxide Nanoparticles Having Long Wavelength Absorbing Chlorins for Highly Enhanced Photodynamic Therapy with Reduced Dark Toxicity

Eun Seon Kang,[a] Tae Heon Lee,[a] Yang Liu,[a] Ki-Ho Han,[a] Woo Kyoung Lee,*[a] and Il Yoon*[a]

Center for Nano Manufacturing and Department of Nanoscience and Engineering, Inje University, 197 Injero, Gimhae, Gyeongnam 50834, Republic of Korea

*E-mail : yoonil71@inje.ac.kr (I. Y.); wlee@inje.ac.kr (W. K. L.)

Characterization Data

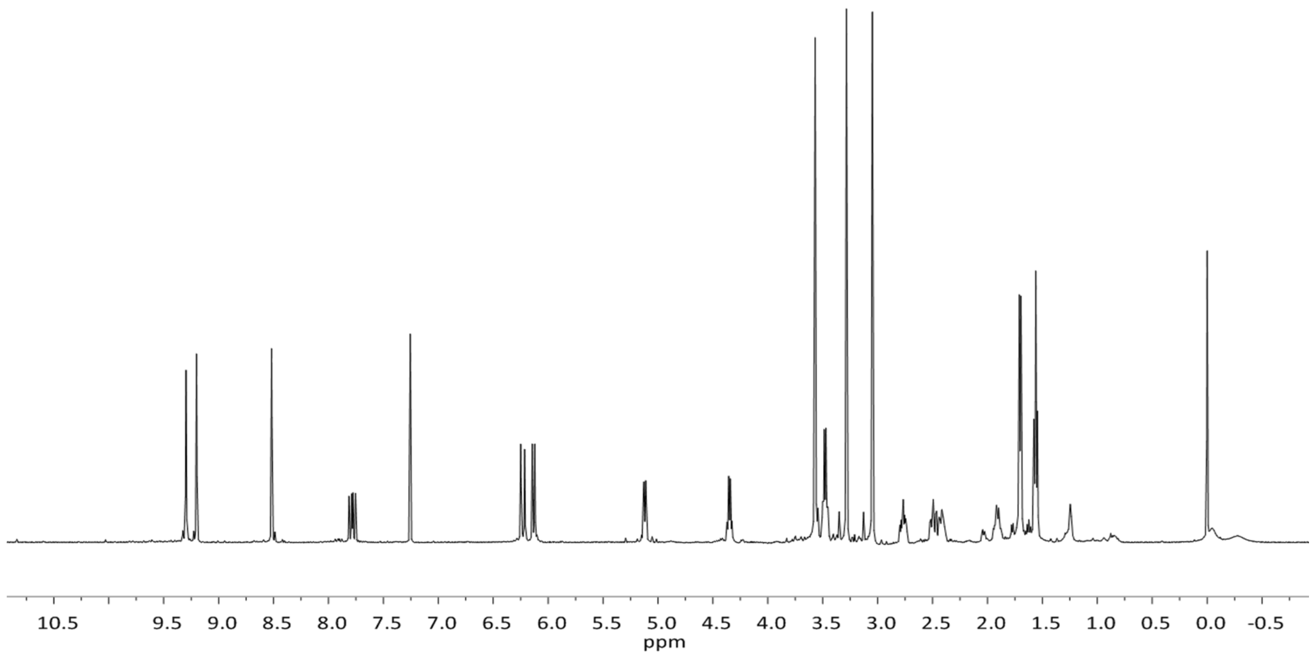

Figure S1. ^{1}H-NMR spectrum of purpurin-18 (P18) (500 MHz, $CDCl_3$, 298 K).

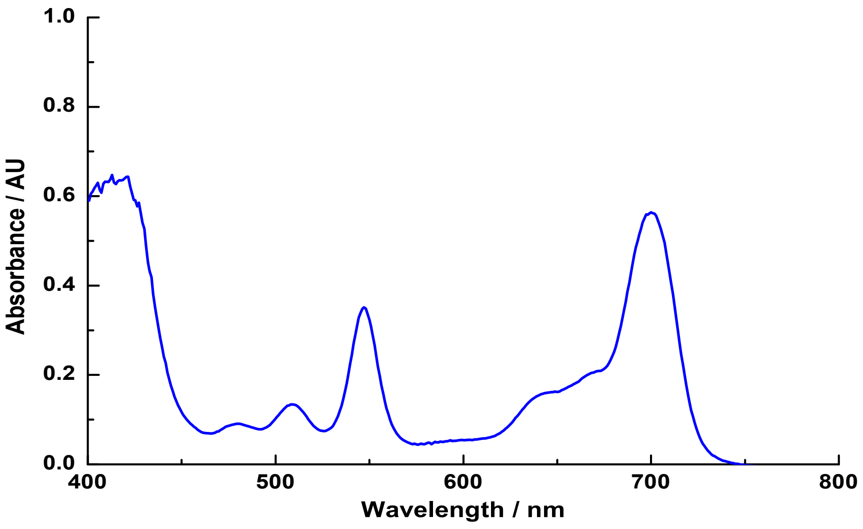

Figure S2. UV-Vis spectrum of purpurin-18 (P18) (CH_2Cl_2).

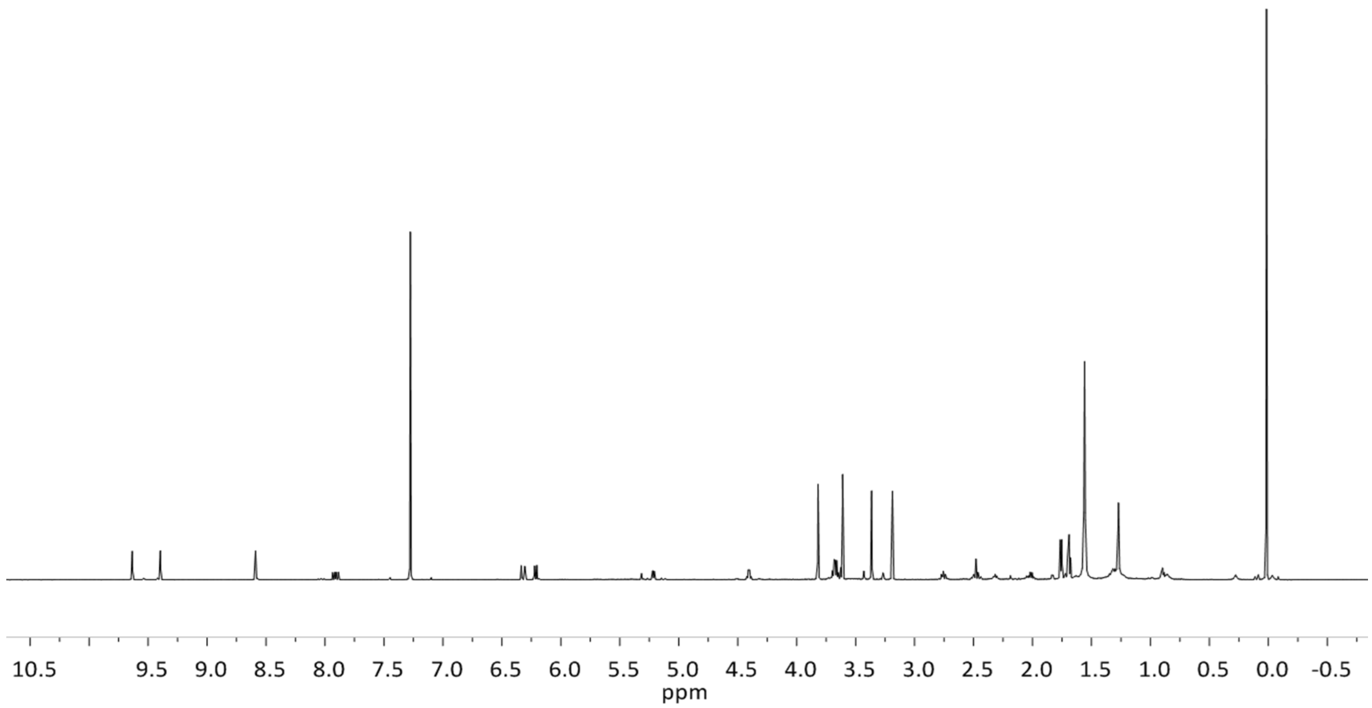

Figure S3. ^{1}H-NMR spectrum of purpurin-18 methyl ester (P18ME) (500 MHz, $CDCl_3$, 298 K).

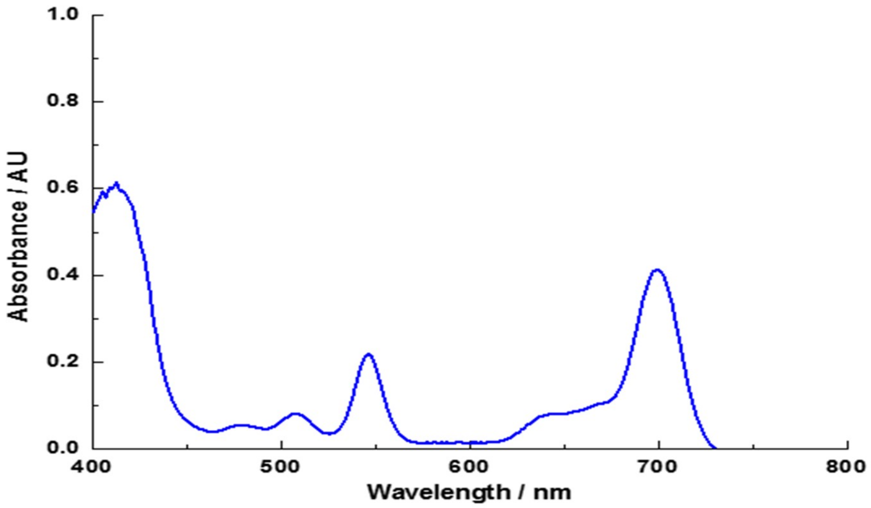

Figure S4. UV-Vis spectrum of purpurin-18 methyl ester (P18ME) (CH_2Cl_2).

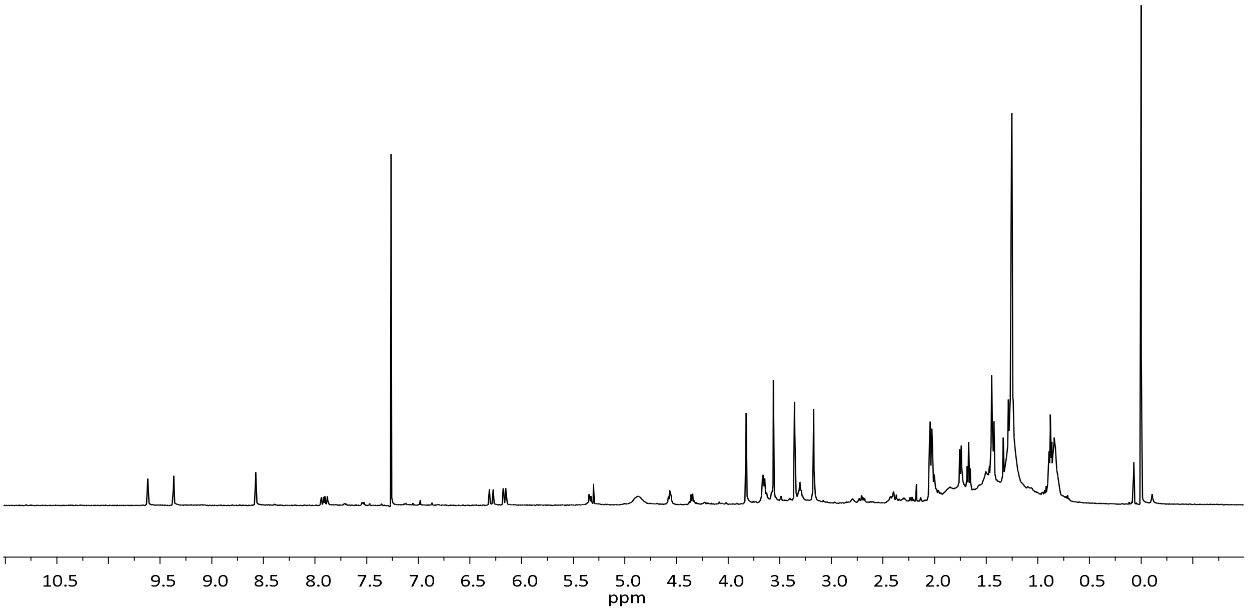

Figure S5. ^{1}H-NMR spectrum of purpurin-18-*N*-ethylamine, PS **1** (500 MHz, $CDCl_3$, 298 K).

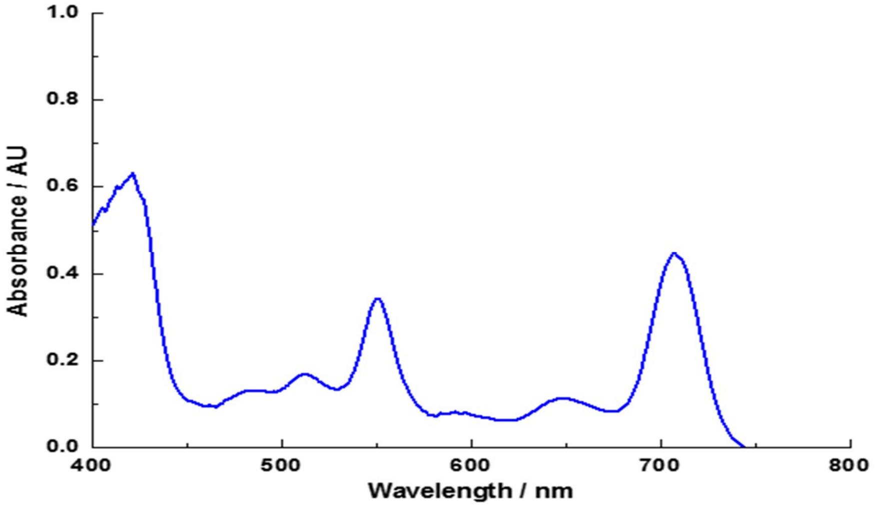

Figure S6. UV-Vis spectrum of purpurin-18-*N*-ethylamine, PS **1** (CH_2Cl_2).

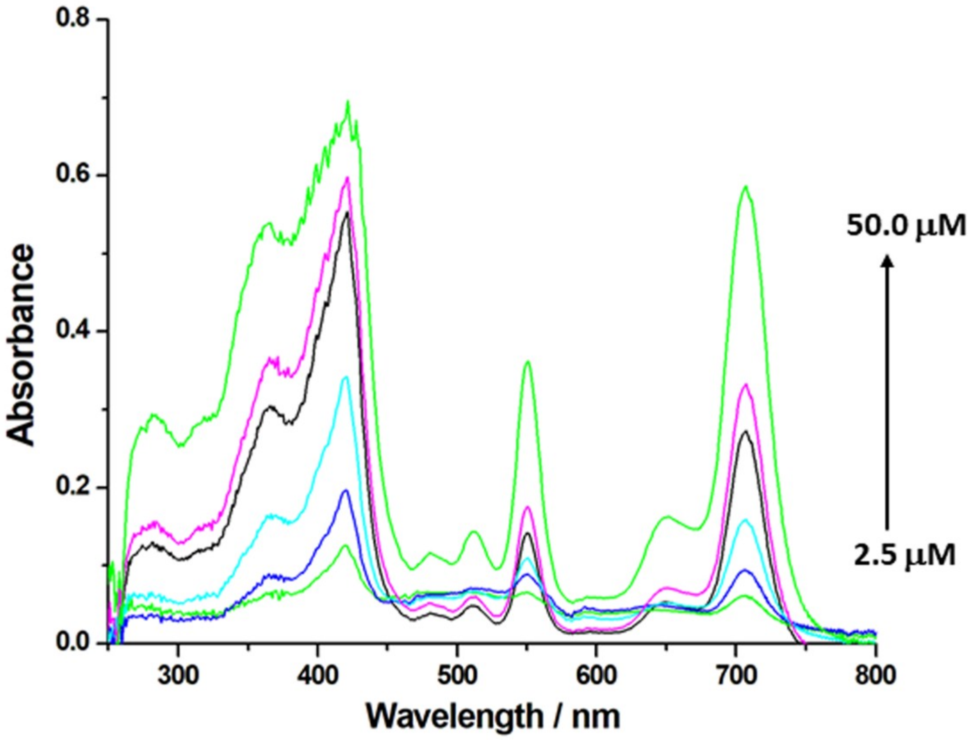

Figure S7. UV-Vis absorbance spectra of PS **1** at various concentrations of 2.5~50 μM (DMSO).

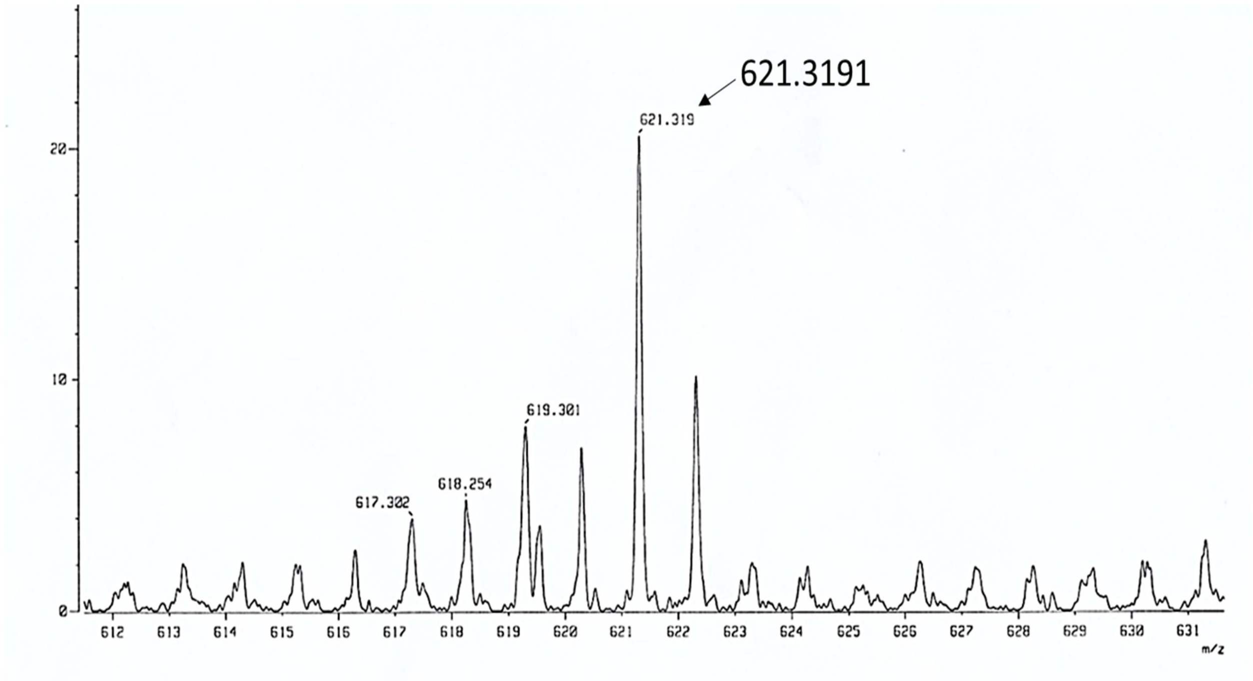

Figure S8. HRFABMS spectrum of PS **1**.

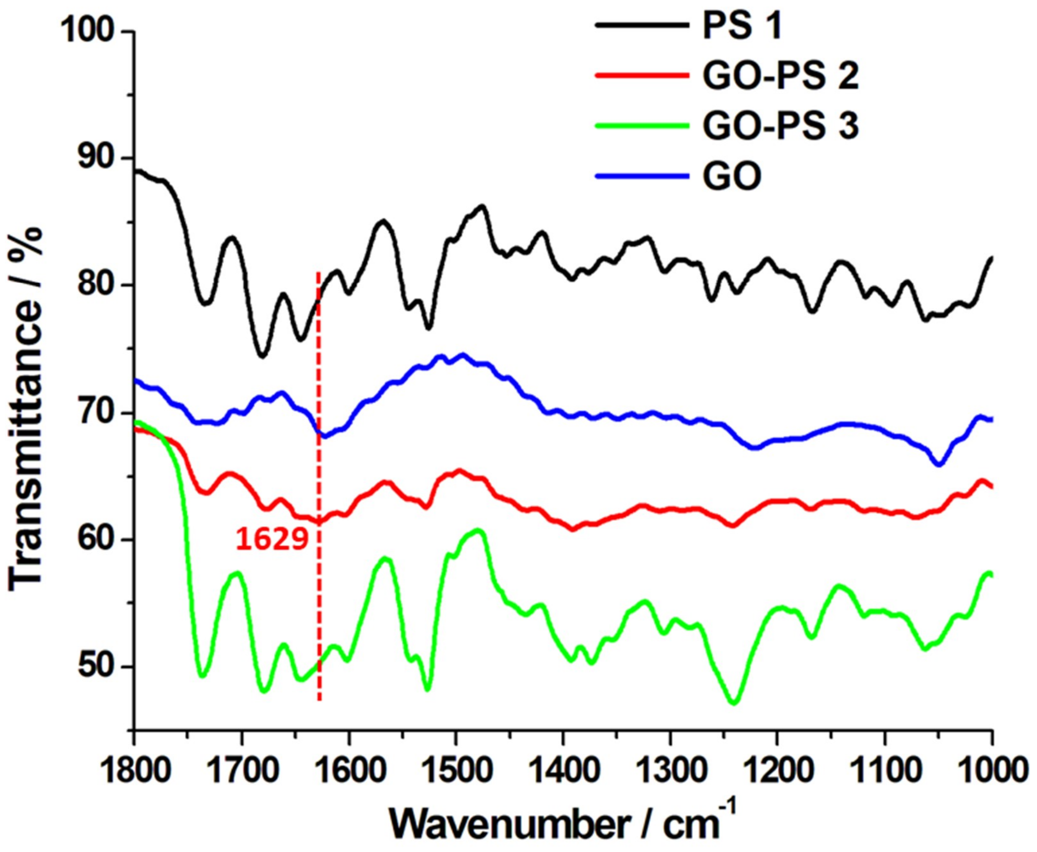

Figure S9. Extended FT-IR spectra of PS **1**, GO–PS **2**, GO–PS **3** and GO at 1800~1000 cm^{-1}.

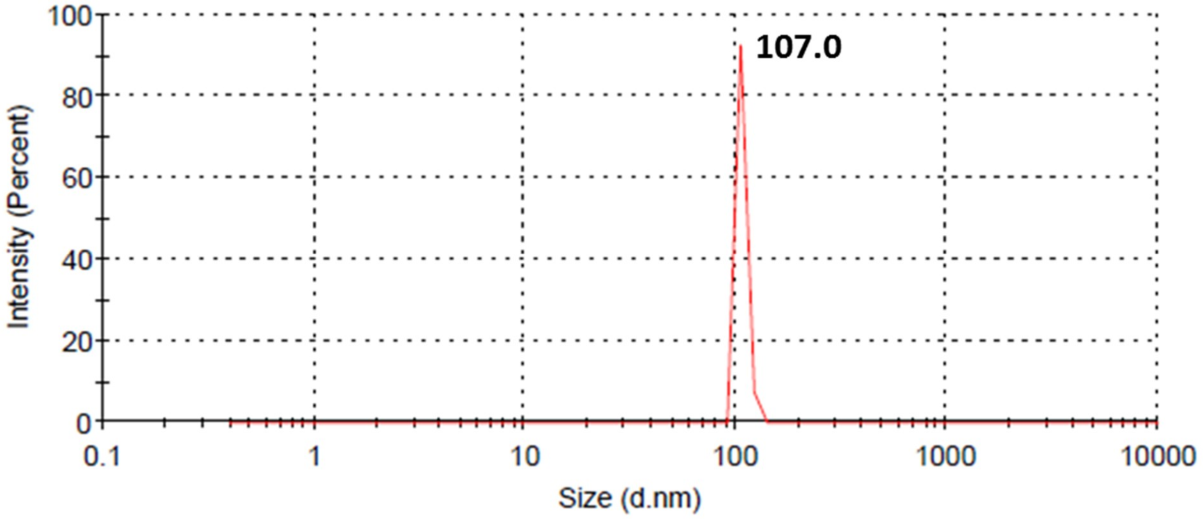

Figure S10. Size distribution of GO measured by dynamic light scattering. Average size is 107.0 ± 5.0.

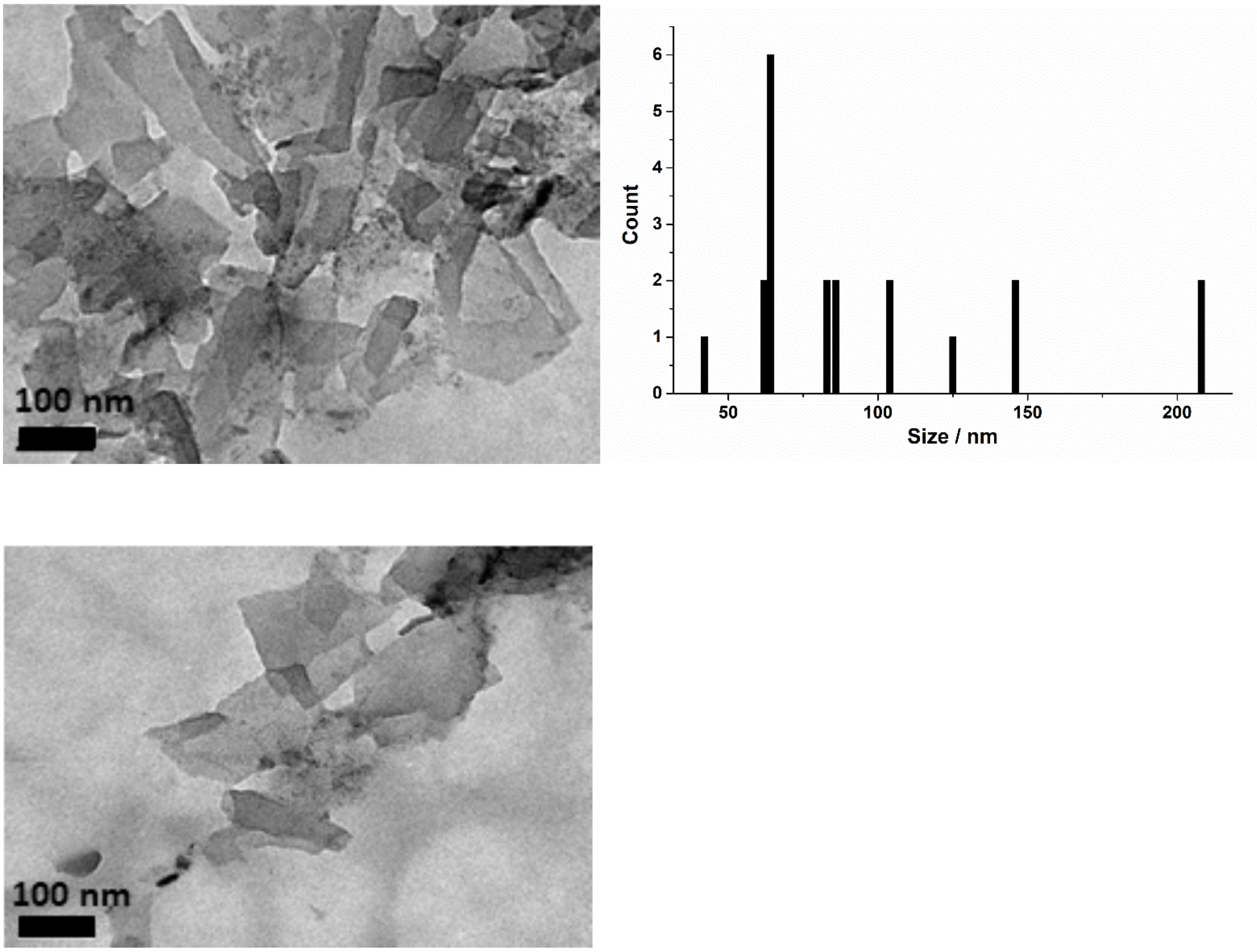

Figure S11. TEM images and size distribution of GO base on TEM images. Average size is 96.1 ± 47.7 nm.

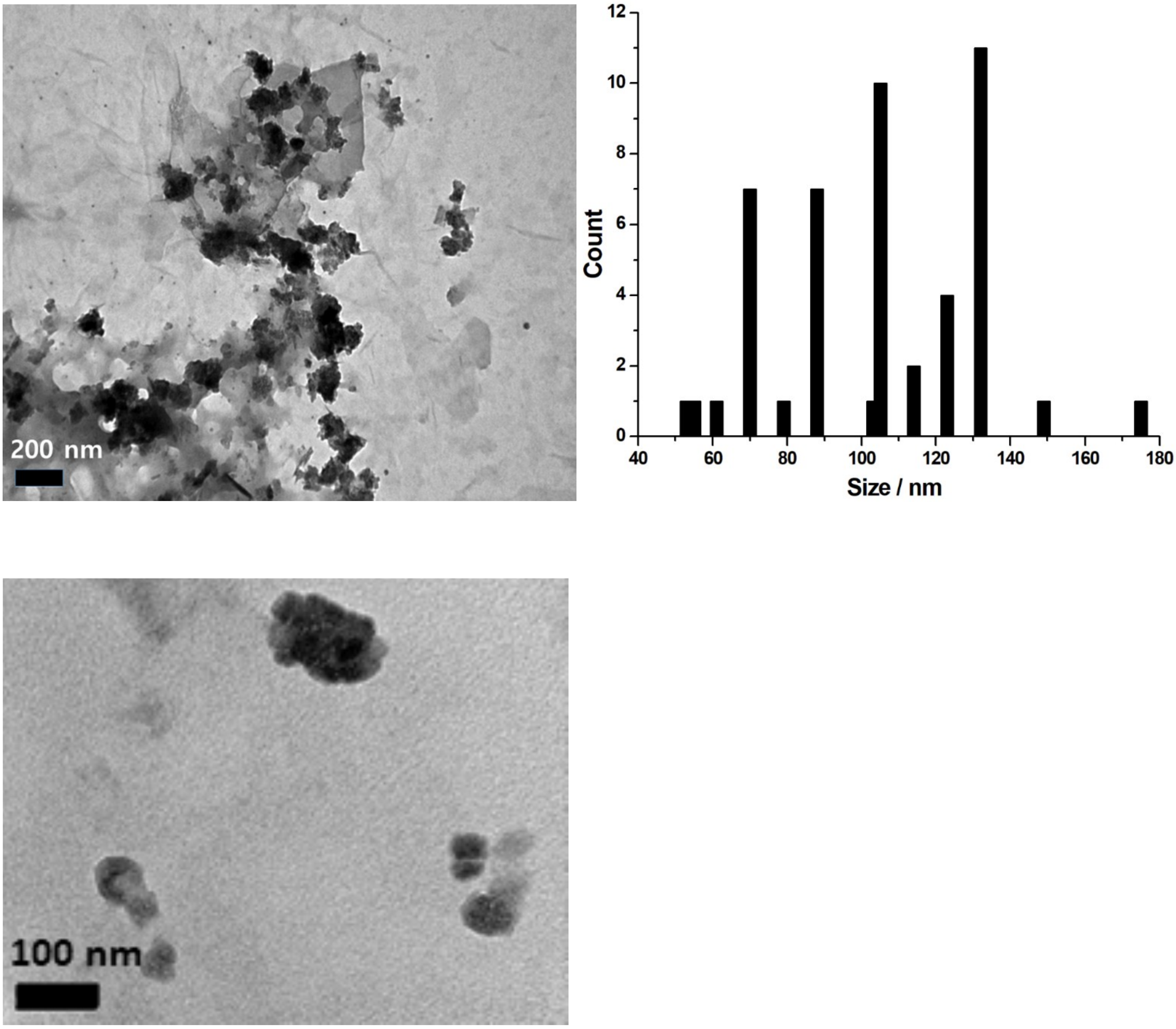

Figure S12. TEM images and size distribution of GO–PS **2** base on TEM images. Average size is 104.2 ± 27.1 nm.

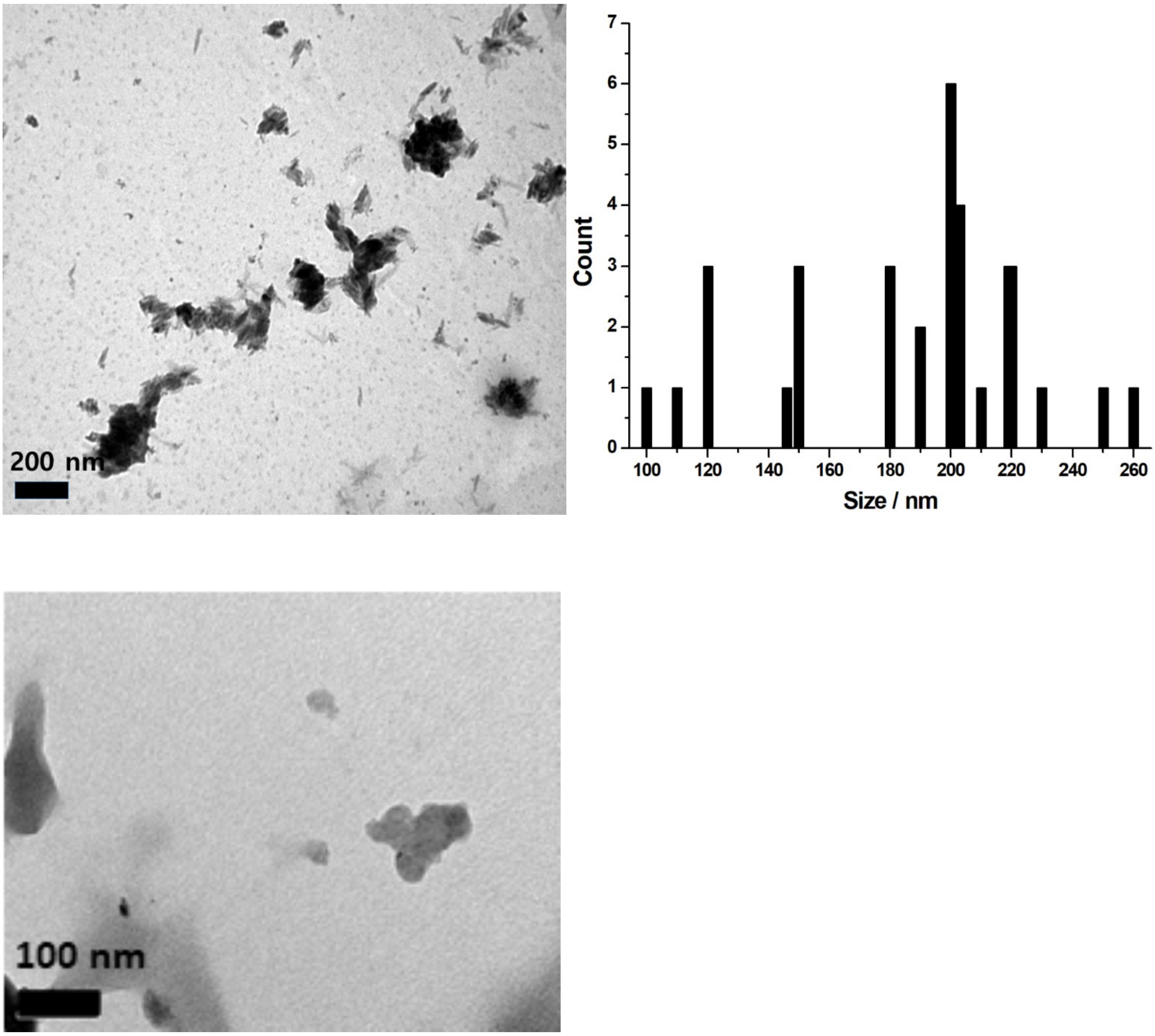

Figure S13. TEM images and size distribution of GO–PS **3** base on TEM images. Average size is 187.2 ± 40.1 nm.

In vitro photosensitizing effect

Table S1. Cell viability (%) of A549 cell only (control), PS **1**, GO–PS **2**, GO–PS **3**, GO(2) and GO(3) for photocytotoxicity against A549 cell lines. The concentration range of PS **1** was 1.0–20.0 µM, and **2** and **3** have a concentration range of 1.4–14.0 µM. The percentage of cell viability was conducted by MTT assay at (a) 3 h, (b) 12 h and (c) 24 h incubation times after irradiation. Error values represent the standard deviation of three replicate experiments.

	Incubation time	
**control**	3 h	100 ± 2.19
	12 h	100 ± 1.22
	24 h	100 ± 5.03

	Incubation time	Concentration (µM) 1.0	2.5	5.0	10.0	20.0
**1**	3 h	100 ± 0.38	98.31 ± 3.40	80.48 ± 1.74	42.40 ± 2.82	21.44 ± 0.30
	12 h	100 ± 1.56	67.86 ± 2.11	36.94 ± 0.38	31.47 ± 1.51	11.96 ± 1.48
	24 h	50.43 ± 3.51	26.10 ± 1.02	19.43 ± 0.83	14.83 ± 0.75	6.72 ± 1.41

	Incubation time	Concentration (µM) 1.4	3.5	7.0	14.0
**2**	3 h	31.56 ± 1.77	24.21 ± 2.59	14.45 ± 1.71	16.50 ± 2.83
	12 h	14.42 ± 0.60	19.13 ± 2.26	10.88 ± 1.15	17.43 ± 3.05
	24 h	16.67 ± 1.67	7.76 ± 0.95	2.96 ± 0.65	2.78 ± 4.31
**3**	3 h	21.73 ± 1.29	21.12 ± 1.60	22.96 ± 0.97	23.85 ± 1.46
	12 h	22.39 ± 1.20	18.90 ± 0.20	15.62 ± 0.35	17.22 ± 0.21
	24 h	29.81 ± 0.66	25.28 ± 0.73	21.14 ± 1.25	20.32 ± 2.71
GO only	3 h	83.89 ± 4.57	83.66 ± 5.65	87.51 ± 3.98	78.76 ± 2.71
	12 h	86.14 ± 5.34	73.12 ± 9.18	75.34 ± 4.20	78.66 ± 5.37
	24 h	84.24 ± 6.72	82.56 ± 2.97	71.86 ± 7.48	67.96 ± 2.78

Table S2. Cell viability (%) of A549 cell only (control), PS **1**, GO–PS **2**, GO–PS **3**, GO(2) and GO(3) for dark cytotoxicity against A549 cell lines. The concentration range of PS **1** was 1.0–20.0 μM, and **2** and **3** have a concentration range of 1.4–14.0 μM. The percentage of cell viability was conducted by MTT assay at (a) 3 h, (b) 12 h and (c) 24 h incubation times no irradiation. Error values represent the standard deviation of three replicate experiments.

	Incubation time	
**control**	3 h	100 ± 3.29
	12 h	100 ± 0.72
	24 h	100 ± 5.21

	Incubation time	Concentration (μM)				
		1.0	2.5	5.0	10.0	20.0
**1**	3 h	100 ± 1.23	100 ± 2.36	100 ± 1.63	100 ± 2.88	83.98 ± 2.28
	12 h	100 ± 0.71	100 ± 2.61	100 ± 4.05	100 ± 0.68	80.89 ± 3.72
	24 h	100 ± 2.19	98.49 ± 1.90	100 ± 1.49	100 ± 3.74	59.72 ± 1.33

	Incubation time	Concentration (μM)			
		1.4	3.5	7.0	14.0
**2**	3 h	100 ± 4.77	100 ± 1.69	89.53 ± 4.70	70.78 ± 2.07
	12 h	91.19 ± 3.28	84.08 ± 4.99	70.50 ± 6.23	56.36 ± 5.92
	24 h	85.89 ± 4.02	78.99 ± 7.30	73.40 ± 6.18	61.22 ± 3.00
**3**	3 h	100 ± 2.24	100 ± 3.37	100 ± 0.77	100 ± 4.10
	12 h	100 ± 1.78	100 ± 3.04	100 ± 0.78	98.06 ± 2.05
	24 h	100 ± 5.44	100 ± 2.31	100 ± 0.77	95.17 ± 3.55
GO only	3 h	82.21 ± 2.75	81.51 ± 4.23	69.02 ± 3.76	65.26 ± 1.00
	12 h	97.77 ± 4.45	95.76 ± 1.74	81.33 ± 3.36	58.57 ± 3.86
	24 h	85.33 ± 6.60	85.92 ± 8.00	74.02 ± 4.87	67.91 ± 1.81

Table S3. Cell viability (%) of A549 cell only (control), GO–PS **2** and GO–PS **3** for photocytotoxicity against A549 cell lines at low concentration. The concentration range of all compounds was 0.14 to 1.05 μM. The percentage of cell viability was conducted by MTT assay at (a) 3 h, (b) 12 h and (c) 24 h incubation times after irradiation. Error values represent the standard deviation of three replicate experiments.

	Incubation time				
control	3 h	100 ± 3.82			
	12 h	100 ± 2.16			
	24 h	100 ± 5.70			

	Incubation time	Concentration (μM)			
		0.14	0.35	0.70	1.05
**2**	3 h	100 ± 3.74	74.54 ± 5.04	47.87 ± 3.45	26.55 ± 2.82
	12 h	100 ± 1.15	56.91 ± 0.70	34.44 ± 2.10	26.41 ± 1.68
	24 h	100 ± 4.55	36.27 ± 7.92	16.01 ± 2.47	11.71 ± 1.17
**3**	3 h	76.35 ± 0.28	1.77 ± 0.45	2.36 ± 1.20	11.50 ± 0.72
	12 h	67.16 ± 1.34	13.02 ± 0.70	12.65 ± 2.82	8.76 ± 4.66
	24 h	67.81 ± 9.78	6.58 ± 0.40	10.75 ± 3.80	6.83 ± 1.39
